# Multifaceted Applications of Ruthenocene and Its Derivatives in Biomedicine, Energy Storage and Electrochemical Sensing

**DOI:** 10.3390/bios16040204

**Published:** 2026-04-03

**Authors:** Ammara Shahid, Sana Sabahat, Aisha Naeem

**Affiliations:** 1 Department of Chemistry, COMSATS University, Islamabad Campus, Islamabad 45550, Pakistan; sp22-pch-003@isbstudent.comsats.edu.pk; 2 Research and Graduate Studies, QU Health, Qatar University, Doha P.O. Box 2713, Qatar

**Keywords:** ruthenocene, bioconjugates, energy storage, redox labelling, anticancer drugs, electrochemical sensing

## Abstract

Ruthenocene (Rc) and its derivatives form a structurally versatile class of metallocenes with unique and multifunctional applicability. This review presents a detailed analysis of Rc chemistry including the structural comparison with ferrocene, its redox behavior, and substituent effects. We also discuss its applications in sensing, energy storage, photochemistry, and biomedicine. Rc exhibits unique conformational and adaptive electronic properties based on one and two-electron oxidation processes. Electrochemical investigations of Rc to date indicate that its redox behavior is strongly dependent on the electrolyte system, exhibiting quasi-Nernstian characteristics, the formation of stabilized dimeric species [Rc_2_]^2+^, and interconversion among Ru(II), Ru(III), and Ru(IV) oxidation states. Rc-based systems exhibit superior performance as redox mediators and labels in electrochemical sensing systems in terms of electron-transfer kinetics, signal amplification, and surface immobilization. In the field of energy storage, Rc decreases the charging overpotential and increases the cycle life of Li-O_2_ batteries. Rc further acts as a photoinitiator via charge-transfer-to-solvent and efficient photoinduced electron transfer in metalloporphyrin and fullerene dyads. In biomedical research, Rc derivatives as well as bioconjugates possess promising anticancer activities, displaying reactive oxygen species generation, topoisomerase inhibition, thioredoxin reductase inhibition, receptor-mediated uptake, and target peptide conjugation. Given its flexible ligand design, electrolyte driven redox behaviors, and antiproliferative properties, Rc exhibits a very adaptive molecular scaffold for next generation electrochemical technologies as well as metallodrug design.

## 1. Introduction

Ruthenocene ((C_5_H_5_)_2_Ru/Rc) belongs to the metallocene class of organometallic compounds. It consists of a ruthenium atom sandwiched between two cyclopentadienyl rings, with the symmetrically aligned ruthenium center attached to the planes of these rings [1]. Unlike ferrocene (Fc), its isoelectronic counterpart, the Rc structure adopts an eclipsed configuration due to the larger ionic radius of the ruthenium atom. The large radius increases the distance between the cyclopentadienyl rings, which in turn reduces steric interactions. In solution, the cyclopentadienyl rings of Rc experience an extremely low energy barrier during rotation [2]. In addition, Rc exhibits high conformational flexibility in solution with cyclopentadienyl rings rotating over a very low energy barrier, resulting in the rapid interconversion of conformations. Consequently, this reflects the dynamic nature of the molecule rather than a fixed three-dimensional arrangement.

**Figure 1:**
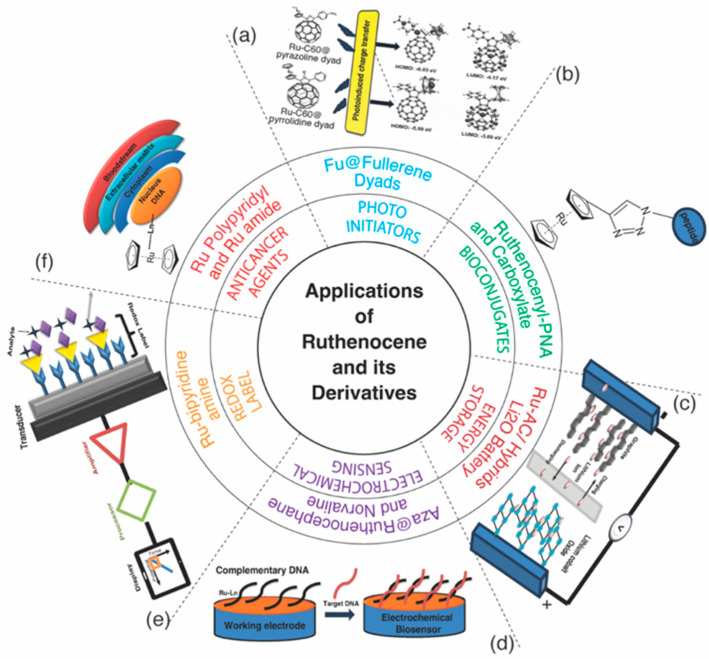
Application of ruthenocene and its derivatives in biomedical and electrochemical areas: (**a**) ruthenocene@fullerene dyads can have a photoinductive effect; (**b**) ruthenocene derivative (Rc-Ln) binds to the DNA molecule in cancer cells to prevent DNA transcription and replication; (**c**) ruthenocene bipyridine complex’s redox label property aids in biorecognition; (**d**) Rc-Ln functionalized working electrode in an electrochemical cell can bind to complementary DNA that will sense target DNA. (**e**) The ability of lithium-ion batteries to charge and discharge can be enhanced by the addition of ruthenocene; (**f**) ruthenocene bioconjugate enhances cellular uptake, stability, and the formation of reactive oxygen species.

The exceptional chemical and thermal stability of Rc contributes to various physicochemical applications [3]. Rc can be utilized independently or conjugated with materials and polymers to enhance its mechanical and optical behavior [2]. It can be used in a number of chemical reactions as a catalyst precursor (Figure 1). Rc undergoes two-electron oxidation in electrochemical systems, which can be influenced by the type of electrolyte solution [3]. In the presence of weakly coordinating anionic electrolytes, it undergoes one-electron instead of two-electron oxidation. This adaptable redox activity makes Rc highly suitable for energy storage and electrochemical sensing applications.

In this article, we review recent advances in Rc chemistry, including its structural comparison with Fc, redox behavior, substituent effects, dimerization mechanisms, energy transfer, biocompatibility, flexible functionalization, and anti-proliferation properties. We also discuss Rc-based templates, anticancer bioconjugates, and photoactive systems for energy storage applications (Figure 1). Furthermore, its applications in sensing, energy storage, photochemistry, and biomedicine are highlighted.

## 2. Structural Comparison of Ruthenocene with Ferrocene

**Figure 2:**
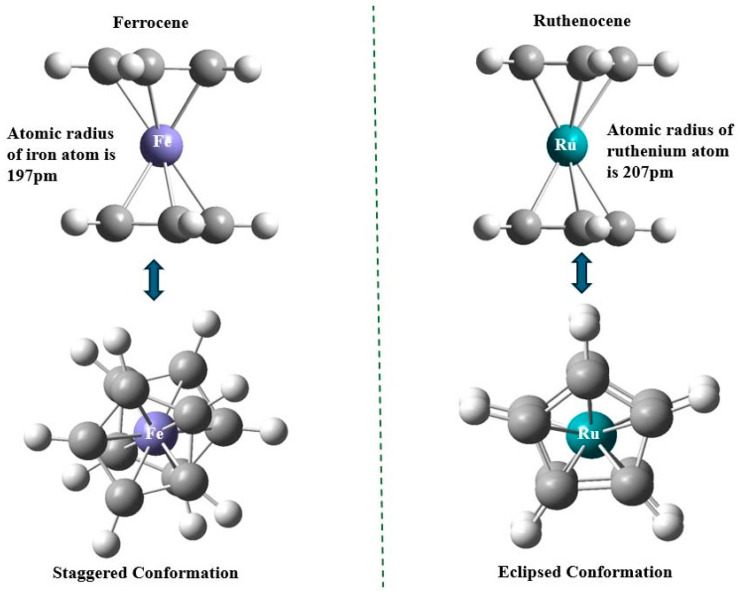
Comparison of electronic structures of ruthenocene and ferrocene.

Rc is structurally comparable to Fc, its metallocene counterpart, due to its stable 18-electron configuration that is not found commonly in other metallocenes [4]. Both Rc and Fc are electronically equivalent except a little structural distinction. The primary distinction is that Rc contains ruthenium as the central atom, whereas Fc contains iron. The difference in metal atoms creates a difference in the atomic radius of the compounds [5]. Ruthenium has a larger atomic radius (~207 pm) than iron (~197 pm) (Figure 2). The larger size of the ruthenium metal in Rc causes longer metal-cyclopentadienyl bond lengths, fewer ring–ring repulsions, and weaker π-overlap, stabilizing the eclipsed form in the solid state [6,7].

Rc adopts an orthorhombic Pnma crystal lattice that remains stable up to about 4 GPa, beyond which irreversible pressure-induced structural perturbations and phonon anomalies are apparent; the molecule has a tendency to adopt an eclipsed conformation and has a moderate rotational energy barrier with respect to the staggered conformation [8,9]. The substitution of ruthenocenyl creates easier electronic interaction and higher metal–ligand coupling than equivalent ferrocenyl analogues, establishing Rc as a better electronic modulator in multimetallic organometallic frameworks [10].

The distinct electronic structures and redox behaviors of Rc and its derivatives are controlled by steric factors (Figure 2). The methyl substitution on the ortho position of ruthenium polypyridyl complexes can have a major effect on the catalysis of CO_2_ reduction through steric interactions [11]. Additionally, dendritic Ru(II) tetramer complexes, which monitor energy transfer in tetranuclear complexes because of the redox behavior of stereoisomers, show indistinguishable absorption spectra and luminescence properties [12].

Structural features significantly influence the oxidation potentials in Rc-terminated oligoenes, which shift to lower values as conjugation increases, indicating stable two-electron redox processes [1]. Because of the steric effects of the cage, solvation, and electron transfer, these systems exhibit wider peak separation and positive shifts in half-wave oxidation potential [13]. The impact of structural tilt on the reactivity of the cyclopentadienyl ring was demonstrated by the oxidation of ring-tilted ruthenocenophanes, which results in stable dicationic dimers containing metal-metal linkages [14]. This is similar to diruthenocenylnaphthalene, which has a distorted structure due to steric interactions between cofacial Rc rings. This distortion is decreased upon oxidation, indicating a drop in electron density on the cyclopentadienyl rings [15]. Together, these investigations show that Rc-based systems exhibit intricate interactions between electronic structure, steric effects, and redox activity.

## 3. Electrochemical Behavior of Ruthenocene and Its Derivatives

Electrochemically, Rc and its derivatives have been extensively studied as redox mediators [1,16,17] and redox labels [18,19,20,21] that can be used to increase the redox potential. Based on these studies, we thoroughly discuss, in detail, such behavior of Rc and its derivatives, highlighting the effects of substituting different ligands, solvents, and supporting electrolyte on the Rc ring (Table 1).

### 3.1. Ruthenocene and Its Derivatives as Redox Label and Mediator

Rc and its derivatives have been studied electrochemically as redox mediators and as redox labels. As a stable redox mediator, Rc was employed to reduce the charging voltage and significantly increase the life cycle of lithium oxide (Li-O_2_) batteries. Rc increases the cycling life of a basic Ketjen black cathode by fourfold, up to 83 cycles [30]. Rc-labeled biomolecules or surfaces have shown excellent immunosensor performance. The electrochemistry of Rc can alter after a target species binds to a surface-bound redox active species. For instance, Rc(acetylacetonate)_2_(bipyridine-NH_2_), when bound by a pentapeptide, causes the formation of a self-assembled monolayer of 6-mercaptohexanoic acid by carbodiimide coupling, demonstrating that Rc(acetylacetonate)_2_(bipyridine-NH_2_) was stable under repeated cycling in biological buffers [18].

Various investigations demonstrate the chemical and electrochemical oxidation of Rc. In the electrochemical oxidation of Rc, the voltage was adjusted to achieve products with the desired oxidation state; usually the oxidation state shifts to +2 or +3 depending upon the applied voltage and desired product. Rc adapts to varying applied potentials and reaction conditions. Denisovich et al. [24] showed that Rc can react chemically and electrochemically with mercury to generate distinct complexes and adducts. Through anodic dissolution at a mercury electrode, the electrochemical technique produces an unstable (C_2_H_5_)_2_Ru^2+^ cation that combines with mercury to form the adduct [(C_2_H_5_)_2_Ru]Hg(BF_4_)_2_ in the presence of BF_4_^−^ ions. Furthermore, Rc can chemically react with mercury halides to form analogous adducts, demonstrating the adaptability of Rc to changing voltage and reaction conditions. In addition to this, when Rc was chemically oxidized in the presence of mercury halides, salts containing ruthenium metal in the +4 formal oxidation state were produced [24].

The electrochemical behavior of diruthenium complexes often involves reversible redox processes, such as the reduction of Ru(III) to Ru(II). Swarts et al. [28] demonstrated reversible one-electron transfer processes for both the ruthenocenyl and ferrocenyl moieties of the β-diketone FcCOCH_2_CORc in both its enol and keto forms under identical electrolyte conditions. In contrast, when the ruthenocenyl moiety was studied in acetonitrile using tetra-n-butylammonium hexafluorophosphate [N(nBu)_4_][PF_6_] as the supporting electrolyte, it underwent oxidation to form Ru(IV) species. These studies show the complex and solvent-dependent electrochemical behavior of bridged diruthenium(II) complexes, demonstrating potential for redox-based applications.

Ferrocenyl and ruthenocenyl derivatives of substituted gold(I) complexes formed with a β-diketone ligand were found to have different electronic settings, with ruthenocenyl derivatives showing a higher level of electronic coupling with the gold center. While ferrocenyl entities involve a reversible one-electron oxidation, ruthenocenyl moieties involve irreversible redox pathways, and therefore suggest that correlation involves greater charge distribution. X-Ray photoelectron spectroscopy (XPS) binding-energy trends also provide further support for the existence of efficient electronic exchange across the multimetallic framework, identifying the role of Rc as a more potent electronic modulator with respect to Fc [49].

### 3.2. Effect of Supporting Electrolyte on Redox Behavior of Ruthenocene

In electrochemical investigations, the supporting electrolyte has a significant impact on the oxidation and reduction processes. The two-electron oxidation of Rc is influenced in the presence of electrolyte [28]. Only one electron is needed for the oxidation process to occur when the electrolyte is composed of weakly coordinating anions such as PF_6_^−^ or BF_4_^−^. Two electrons from Rc are transferred to an external acceptor during oxidation in the presence of strongly coordinating anions such as I^−^, Cl^−^, or Br^−^. In the presence of a supporting electrolyte with a weakly coordinating anion, the redox reaction is reversible, while in a strongly coordinating anionic environment, the reaction will be irreversible due to the formation of the ruthenocenium complex [18].

Rc exhibits varied properties in aqueous and non-aqueous solvents. Non-aqueous solvents such as acetonitrile and dichloromethane are frequently used in electrochemical investigations because they provide a much more stable environment and produce more consistent and reproducible results (Table 1). However, using aqueous media may cause difficulties due to hydrolysis and other side reactions [27]. When choosing supporting electrolytes in aqueous solvents, the redox behavior of Rc can be significantly affected. Although hydroxylation is more likely to occur in basic solutions, protonation can also happen in acidic solutions [27]. Tetra-n-butylammonium hexafluorophosphate is an example of a supporting electrolyte that can impact the oxidation behavior of Rc because of the reaction of the PF_6_^−^ anion with RuCp_2_^+^. In aqueous environments, this typically has minimal impact.

Electrolytes and non-aqueous solvents provide wider potential windows, allowing for the study of redox processes that may not be accessible in aqueous solvents [27]. The peak current and peak potential of Rc are similarly impacted by temperature variations of the supporting electrolyte. A temperature increase typically causes the activation energy barrier to be overcome, the kinetic rate to increase, reversibility to occur, and the rate of diffusion to increase. For instance, Rogers et al. [29] used six distinct ionic liquids, kept at room temperature with differing viscosities to study the electrochemical behavior of Rc at various concentrations and temperatures. Chronoamperometry indicates that Rc oxidation occurs via a two-electron mechanism, involving the one-electron oxidation of Rc to the [Rc]^+^ monocation, followed by dimerization to generate the dimeric bis(η^5^-cyclopentadienyl)ruthenium(II) cation [Rc_2_]^2+^, as has been speculated (Table 1).

The solvent-dependent redox behavior of Rc derivatives with β-diketonato titanocene complexes was investigated by using acetonitrile and dichloromethane with 0.1 M [NBu_4_][B(C_6_F_5_)_4_] as the supporting electrolyte. The variation in solvent significantly affected the redox potential of the complexes [47]. The ruthenium redox center Ru(IV) was involved in the electrochemically and chemically irreversible two-electron transfer redox couple, Rc/Rc^+2^ in dichloromethane/0.1 M [N(nBu)_4_][PF_6_], while the TiIII/TiIV and ruthenocenyl couples displayed irreversible electrochemical behavior in acetonitrile/0.1 M [N(nBu)_4_][PF_6_]. The range of the redox potentials for the TiIII/TiIV, Rc/Rc^+1^ and Rc/Rc^+2^ couples was 2.30-fold for both solvents, 1.02-fold for dichloromethane, and 1.37-fold for acetonitrile, respectively [47]. The anionic effect of pentafluorophenylborate as a supporting electrolyte in an electrochemical system was investigated in a study [42]. In the presence of [N(nBu)_4_][B(C_6_F_5_)_4_], anodic processes can be electrochemically resolved more effectively than in [N(nBu)_4_][PF_6_]. Electrochemical processes involving cationic analytes can benefit from changing the supporting electrolyte to weakly coordinating anions such as [B(C_6_F_5_)_4_] anions. When dichloromethane/[N(nBu)_4_][B(C_6_F_5_)_4_] was used as a supporting electrolyte, redox reactions involving positively charged analytes were favored. In such cases, the strong reducing conditions can cause the analyte to become positively charged after the process of reduction, which enhances the redox signal of tetrahydrofuran [42] (Table 1).

Substitution of [3,5-bis(trifluoromethyl)phenyl]borate affects the electrochemical response of Rc reported by Hill et al. [25]. In this study, tetrabutyl-ammonium tetrakis [3,5-bis(trifluoromethyl)-phenyl]borate (TBATFPB) in dichloromethane was used to investigate the electrochemical response of Rc. In contrast to earlier research [18], in which Rc exhibited an irreversible two-electron oxidation with other supporting electrolytes, Rc displayed quasi-reversible one-electron oxidation in 0.1 M TBATFPB in dichloromethane solvent. The Rc oxidation process was more reversible because the TBATFPB electrolyte is non-coordinating and suppresses secondary reactions like dimerization and nucleophilic attack [25].

In addition to the supporting electrolytes discussed above, the size of the anion in solution has a significant impact on the redox behavior of Rc. It was explored that the oxidized ruthenium ion can form a charge-transfer in combination with sodium toluene sulfonate. The distinction in anionic size produces distinct voltammetric profiles, and the reaction of dissolved Rc in non-aqueous fluids is largely dependent on the electrolyte in solution [37]. This phenomenon can be seen in the electrochemical reaction of 0.1 mM RuSO_3_ dissolved in three different electrolyte solutions, namely 0.1 M potassium chloride, 0.1 M potassium hexafluorophosphate, and 0.1 M sodium toluene sulfonate. These electrolytes were strong and fully dissociable, making it clear that the response was dependent on the anion rather than ionic strength (Table 1). The results showed that the electrochemical response of 0.1 mM RcSO_3_^−^ dissolved in 0.1 M NaCl was similar to the response in 0.1 M potassium chloride, suggesting that the anion in solution was the only factor influencing the voltammetric signal of RcSO_3_^−^. Two oxidation peaks were seen at +0.85 V and +0.97 V in the presence of potassium chloride and potassium hexafluorophosphate, with higher currents seen at +0.97 V in the presence of potassium chloride [37].

The nature of the anion and solvent has a significant impact on the diffusion rate of Rc in a polymer matrix. Shimura et al. [32] outlined the method by which anions and solvents coordinate with the Rc sites in the polymer matrix and in solution. Even octamethylruthenocenophane, a structurally constrained Rc, undergoes completely irreversible oxidation in acetonitrile when it is in solution [32]. Conversely, the surface of the polymer matrix immobilized with Rc produces an electrochemical response that exhibits a re-reduction peak in acetonitrile. This suggests that compared to the solution, the polymer matrix exhibits a significantly slower rate of solvent and electrolyte anion coordination. It was anticipated that the concentration of redox species and coordinating ligands (solvent/electrolyte anion), diffusion rate of ligands, and the coordination kinetics would be the main factors influencing the overall coordination rate (Table 1) [32].

In non-aqueous solvents, the electrochemical behavior of Rc is sensitive to solvents and electrolytes. Swarts et al. [28] observed a quasi-Nernstian reaction in the electrochemical oxidation of Rc in dichloromethane with the supporting electrolyte [B(C_6_F_5_)_4_]^−^(TFAB) or [B(C_6_H_3_(CF_3_)_2_)_4_]^−^(BArF_24_). In the reaction condition at lower temperatures, a metal–metal coupled dimer [Rc_2_]^2+^ was preferred over the ruthenocenium ion (+1). This was often the outcome of self-reactions in the absence of nucleophiles that produce Rc–Rc or Rc–C bonds in the presence of dry and non-donor solvents. The oxidation of Rc was intrinsically a one-electron reaction, as the ruthenium ion was a more potent one-electron oxidant due to its higher positive E_1/2_ value of 0.41 V in the dichloromethane/[NBu_4_][TFAB] electrolyte [28].

Trupia et al. [26] examined the effect of electrochemically oxidizing Rc in the presence of bulky anions [NBu_4_]A, where A = [B(C_6_F_5_)_4_]^−^ or [B(C_6_H_3_(CF_3_)_2_)_4_]^−^ in dichloromethane as the solvent. This produced the dimeric indicator [Rc_2_]^2+^, which was in equilibrium with the 17-electron ruthenium ion +1. The quasi-Nernstian cyclic voltammetry behavior was used to explain the dimeric indicator, dimeric bis(η^5^-cyclopentadienyl)ruthenium(II) cation [Rc_2_]^2+^, with E_1/2_ being 0.41 V at room temperature. Bulk electrolysis at 243 K and cyclic voltammetry offer direct electrochemical evidence for [Rc_2_]^2+^. In the dimeric bis(η^5^-cyclopentadienyl)ruthenium(II) cation, a highly irreversible two-electron cathodic reaction occurs at an Ep_c_ of around 0 V. Half of the original Rc was regenerated through the anodic electrolysis of Rc at 243 K and the cathodic electrolysis of [Rc_2_]^2+^; employing tetra(pentafluorophenyl)borate [B(C_6_F_5_)_4_]^−^ as the supporting electrolyte causes precipitation, which facilitates the isolation of [Rc_2_] dimeric bis(η^5^-cyclopentadienyl)ruthenium (Table 1) [26].

The anionic size, coordination efficiency, and the choice of supporting electrolyte impacts the redox behavior of Rc and its derivatives [18,42]. The type of electrolyte, solvent, and anion can alter the oxidation state, reversibility, and stability of Rc-based compounds. Non-aqueous solvents, particularly acetonitrile and dichloromethane, tend to offer better stability and wider potential windows, making them ideal for electrochemical studies [37]. In contrast, aqueous media may introduce challenges such as hydrolysis and unwanted side reactions. Changes in composition by using supporting electrolytes with different anionic substitutions, [N(nBu)_4_][B(C_6_F_5_)_4_] or [N(nBu)_4_][PF_6_] in an electrolytic system might significantly affect the redox potential and reversibility of Rc derivatives [27]. Similarly, larger anions, such as [B(C_6_F_5_)_4_]^−^, have been shown to be particularly effective at resolving anodic processes and increasing the reversibility of redox reactions in electrolytes [26]. These studies show that the utilization of Rc in a variety of electrochemical settings is attributed to its ability to coordinate with various anions and solvents.

### 3.3. Effect of Substituents on Redox Behavior of Ruthenocene

Substituents with electron-donating and withdrawing properties have a substantial influence on the redox potential of Rc when attached to a cyclopentadienyl ring of Rc [50]. Electron-withdrawing substituents such as the benzoyl group in benzoylRc decrease the ease of oxidation, while electron-donating substituents such as 1,1-dimethyl in 1,1-dimethylRc cause facile oxidation, according to the oxidation–reduction potentials measured by chronopotentiometric methods at a platinum electrode in an acetonitrile solution (Table 1). These trends are consistent with the expected inductive effects [22].

The effect of the number and type of linker groups on oxidation potential was studied electrochemically by Ismail et al. [48]. The study was conducted on a chiral organometallic nucleoside analogue that contained Rc with alkylthymine and alkylhydroxyl groups connected to a single cyclopentadienyl ring [48]. Their oxidation potentials were examined by using cyclic voltammetry, which demonstrated the impact on oxidation potential due to the type and number of linker groups such as alkylthymine and alkylhydroxyl groups affixed to Rc. The charged ruthenium ion achieved greater stability as the number of electron-donating groups on the cyclopentadienyl ring increased [48].

The two-electron Rc/Rc^2+^ redox couple, which showed chemical and electrochemical irreversible behavior, was investigated while studying chalcones such as Rc–CO–CH@CH–Ph, Ph–CO–CH@CH–Rc, and Rc–CO–CH@CH–Rc in acetonitrile with 0.1 M [N(Bu)_4_][PF_6_] as the supporting electrolyte [43]. It was found that oxidizing chalcones with a carbonyl group adjacent to Rc was more difficult because of the electron withdrawing-effect of the carbonyl group. Furthermore, the position of Rc in RcCOCH@CHRc affected the reduction potential of the electroactive ruthenocenyl molecule, depending on its adjacency to the double bond or carbonyl group [43].

A research study based on hemilabile O,N-chelating ligands attached to Fc- and Rc-based bis-palladacycles resulted in enhanced bimetallic catalytic activity. This was revealed by structural studies in which μ_2_-(O,N) coordination caused reduced efficiency, whereas κ^2^-(O,N) chelation restored and improved catalytic performance [51]. Rc-containing β-diketones of the type RcCOCH_2_COR, with R = C_10_F_21_, C_6_F_5_, and C_10_H_21_, were investigated electrochemically in the non-interacting solvent dichloromethane and 0.1 M [N(nBu_4_)][B(C_6_F_5_)_4_] electrolyte [44]. The results showed electrochemically irreversible one-electron transfer Rc/Rc^+^ couplings in the potential range 650 < Ep_a_ < 1110 mV. The first-order rate constant of the enol to keto conversion varied between 220 and 50,000 s^−1^ based on the R-groups and the deuterated solvent (chloroform/acetonitrile), and the enol isomer of the fluorinated β-diketones was more than 90% abundant. This is evident from the fact that compounds with electron-withdrawing R-groups had the highest oxidation potentials, while compounds with electron-donating R-groups showed the lowest potentials [44].

A Rc-containing methacrylate monomer with tetrabutylammonium hexafluorophosphate (TBAPF_6_) as the supporting electrolyte showed reversible redox reaction [45], while the redox process of a ruthenium complex containing the pentamethylcyclopentadienyl ligand (PMAERc) was not reversible, with the oxidation peak at 1.13 V and the reduction peak almost undetectable. An increased ion pairing of cationic ruthenium with PF_6_^−^ reaction could be linked to the irreversible reduction of Ru(III) to Ru(II). Additional supporting electrolytes with different anions (NO^−3^, Cl^−^, and BPh_4_^−^) and solvents (dichloromethane and dimethylformamide) were tested in order to explore the detailed electrochemical behavior of PMAERc. They all showed irreversible redox behaviors at different scan rates [45].

The increase in silyl substitution (SiMe_3_/SiMe_2_H) on the cyclopentadienyl rings of Rc increases the stabilization of unusual coordination geometry and oxidation states while simultaneously enhancing solubility in non-polar solvents and allowing for an increase in volatility [52]. Silylated ferrocenyl and ruthenocenyl derivatives, including pentasilylated cyclopentadienyl derivatives, were obtained from perhalogenated precursors and validated the extent of silyl substitution as well as its impact on Rc frameworks [52]. Various Rc moieties can be incorporated at polymer backbones as pendant substituents for the synthesis of organometallic polymers with tunable physical properties.

**Figure 3:**
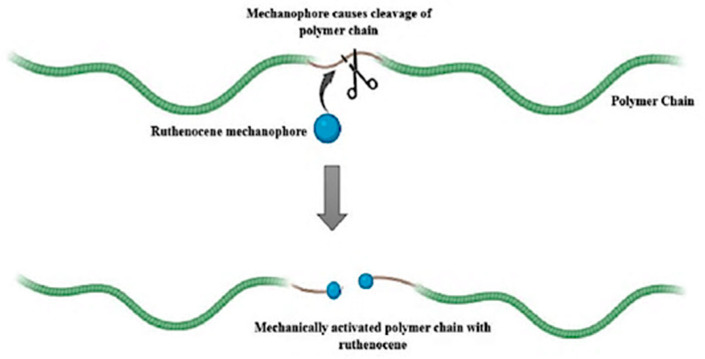
Ruthenocene-containing polymers exhibit mechanoresponsive activity under mechanical stress, resulting in chain scission.

The photophysical, thermal, and electrochemical properties of these materials are dramatically influenced by the spatial association of the Rc units with the polymer chain while the inherent redox activity and structural stability of the Rc entity remain unchanged. Sha et al. [53] showed the possibility of precisely synthesizing Rc-functionalized homopolymers as well as more complex architectures, such as random and block copolymers, by using living ring opening metathesis polymerization (ROMP), were conserved. These observations highlight the usefulness of Rc-based polymers as modifiable platforms for the development of functional materials with controllable metal-dependent properties [54]. Rc-based polymer mechanophores can release activated species such as radicals, zwitterions, and carbenes in response to mechanical stress [55,56,57]. This mechanochemical activation is strongly influenced by the shape and composition of block copolymers; higher glassy block content enhances activation at higher stresses but lower strains [58] (Figure 3). The discovery of Rc as a potential stress-responsive building block for Rc-based polymers has further expanded the range of potential applications [59,60].

The oxidation of ferrocenylruthenocenyl-methanes and Rc has been investigated at dropping mercury and Pt-rotating disc electrodes (Table 1). It was found that the reversible one-electron oxidation of Rc and their derivatives at the dropping mercury electrode decreased the rate of oxidation. A correlation study between Rc and its derivatives showed that, mostly via an inductive mechanism, Rc derivatives transfer the electronic effects of substituents to the ruthenium atom [23]. Moreover, investigating the effects of different electrode materials or surface modifications on the redox behavior could offer valuable insights into improving electron transfer kinetics, with potential applications in electrochemical devices.

## 4. Ruthenocene and Its Derivatives as Electrochemical Sensors

Rc-modified electrodes offer several advantages in terms of electrochemical sensing. It boosts electron mobility and can be used to promote electron transport between biological molecules and electrodes, resulting in stronger signals [61]. The surface of Rc and its derivatized complexes can be functionalized by chemical modification of the electrode surface, which allows for rapid and easy electron transfer between analytes. It also increases the compatibility and selectivity of the surface to attach the analyte, which results in greater sensitivity, as shown in Figure 1 [62]. Due to advancements in sensing technology, Rc has not only used in electrochemical biosensing through electrode modification, but also combined with other nanomaterials for electrochemical applications. The resulting nanocomposites possess greater surface area for the immobilization of biological analytes, consequently increasing sensor sensitivity [62].

Rc derivatives can also be combined with biomolecules such as antibodies or DNA probes to build sensitive and selective biosensors [63]. Upon binding to the biosensor, Rc initiates a reversible oxidation–reduction reaction, which results in the production of an electrical signal proportional to the analyte concentration. In biomedical diagnostics, Rc-based sensors have been developed to detect important biomolecules. For example, a ruthenium-mediated photoelectrochemical sensor based on molecularly imprinted polymers was developed for detecting bisphenol A, achieving a linear range of 2–500 nM and a detection limit of 1.2 nM in biological samples, highlighting the potential of ruthenium-based sensors for food quality monitoring [63].

Due to their tunable redox behavior and efficient electron-transfer characteristics, Rc-based systems have been widely explored in chemical and biosensing applications. Selective cation sensing, both optically and electrochemically, was enhanced by intramolecular electron-transfer interactions between homo- and heterometallic Fc-Rc triads [64]. Spectroelectrochemical studies revealed low-energy near-infrared bands in a molecule with a central Rc unit and two peripheral Fc groups, indicating strong intramolecular charge transfer between Rc and Fc and efficient long-range electron transport. These triads therefore function as selective optical and dual redox chemosensors for Hg^2+^, Pb^2+^, and Zn^2+^ [65]. In another sensing application study, researchers created a polypyrrole film-based photoelectrochemical immunosensor that contained biotinylated-Rc for antibody detection [66]. The antibody detection platform was developed by the biotinylation of cholera toxin and avidin onto the film. The addition of cholera toxin antibodies led to a decrease in photocurrent, which resulted in quantitative detection over a concentration range of 0–200 mg mL^−1^ [66]. Furthermore, a nitric oxide sensor based on [Rc(bpy)_2_(dabpy)]^2+^ enabled spectrophotometric and fluorescence-based detection of nitric oxide in a concentration-dependent manner, including in endothelial cell models, where the presence of exogenous nitric oxide produced a ~5.2-fold increase in fluorescence compared with the baseline levels [67].

Rc derivatives can be used to develop optical sensors. The addition of Rc to EuCl_3_ complexes produces luminescence, which can modulate the ECL emission based on the target analyte binding. The identification of a specific analyte was made possible by the modulation of the emission properties of Rc [68]. The formation of Eu(III) complexes with Rc can help build electrochemiluminescence sensors and provide a more sensitive detection approach (Table 1) [68].

The mixed aza-substituted metallocenophanes, with a [2.2] and [3.3] Fc and Rc multifunctional molecular system, have potential in investigating the intramolecular transfer of charge and recognition in metal-based mechanisms. These combined metallocenes produced a localized excitation band in the near infrared that was indicative of intramolecular charge transfer via carbodiimide bridges connecting the Rc and Fc units [38]. In addition to the exceptional cation-sensing properties of mixed diaza [2.2]ferroceno-ruthenocenophanes, it showed high selectivity for Zn^2+^ ion detection. When Zn^2+^ ions were present, the metal–ligand transition band in the absorption spectra of these compounds was shifted by 100 nm [38].

To develop molecular sensors for the detection of specific ions, polyanionic biomolecules, and to facilitate redox catalysis and bioinorganic recognition processes, supramolecular host–guest macrocyclic receptor molecules were designed by attaching redox active ionophores (guests) to the host’s molecular cavities. Yin et al. [33] described the design, characteristics, and chemical framework of a new polyazamacrocyclic receptor combining three redox active units in acetonitrile solvent and Bu_4_NBF_4_ as the supporting electrolyte, revealing an irreversible reaction (Table 1). The anodic potential was more positive than the Rc anodic wave (0.78–0.80 V), indicating that the oxidation of Ru(II) to Ru(III) accounted for the observed irreversibility.

The redox properties of ruthenium complexes containing ONO pincer frameworks and ruthenium-complex-bound norvalines are strongly influenced by Rc(pydc)(terpy) and Rc(pydc)(tBu-terpy) units [46]. The electron-doping of the tBu-terpy ligand was responsible for stabilizing the Ru(III) state, resulting in a larger shift in the oxidation potential than the terpy ligand. Incorporation of amino acid moieties further alters the redox behavior of these complexes, indicating that even minor structural modifications can significantly affect the electronic environment and electrochemical properties of ruthenium-based systems [46].

Rc terminated dyads have been studied as redox active, optically responsive systems capable of intramolecular electron transfer and selective cation sensing, with practical optical detection potential and metal-ion recognition. The electrochemical, electrical, and cation sensing characteristics of Rc-terminated 2-aza-1,3-butadiene open and closed dyads were examined for intramolecular electron transfer and metal recognition mechanisms. Rc showed a quasireversible oxidation (ΔEp ≈ 0.11 V), which is useful redox behavior for sensing [36]. The presence of low-energy bands in the near-infrared region in the monooxidized forms suggests the presence of optically-induced interconversion transitions between the ruthenium and iron redox centers. These systems act as optical sensors for multiple cations such as Ni^2+^, Zn^2+^, Cd^2+^, Hg^2+^, and Mg^2+^ by color shifts. In the presence of Ca^2+^ cations, the new low energy band causes a color shift that can be utilized to detect Mg^2+^ cations with the unaided eye [36].

In general, Rc-based systems are more suitable than Fc for sensing applications in chloride-rich environments, as ferrocene becomes unstable in the presence of chloride ions due to its oxidation to the ferrocenium species. For example, ruthenium(II) acetylacetonate bis(2,2′-bipyridine-4-ylamino), [Rc(acac)_2_(bpy-NH_2_)], has been shown to replace Fc in chloride-based sensing applications for this reason [56]. The comparative summary of the electrochemical and mechanistic differences between Fc and Rc relevant to sensing applications is given in Table 2. The electrochemical parameters are from the literature provided in the manuscript [45,46,47,48,49,50,51,52,53,54,55,56,57]. The values may differ on the basis of substitution patterns and electrolyte conditions.

[Rc(acac)_2_(bpy-NH_2_)] exhibits high stability when bonded to a gold electrode surface because it is highly sensitive for detecting chloride. The invention of this homobifunctional redox label can be used to design a range of immunosensors and biosensors for application in chloride-containing fluids such as human serum [69]. The redox species were found to be extremely responsive to surface coupling reactions and stable during repeated cycling in biological buffers. This was established by connecting a pentapeptide to the redox label after it had been fixed onto a self-assembled monolayer of 6-mercaptohexanoic acid by carbodiimide coupling [18].

These applications highlight the versatility and effectiveness of Rc-modified electrochemical sensors across various fields, particularly in biomedical diagnostics and metal-ion monitoring. All of these studies suggest that the role of Rc in enhancing sensing performance lies primarily in improving redox activity rather than merely increasing the electron-transfer kinetics.

## 5. Ruthenocene as Active Monomolecular Template and Stable Dimeric Complex

Rc complexes have the potential to serve as active monomolecular templates for electrochemical surface studies. Weidner et al. [41] studied the dicationic bridged REBTA (Ruthenium ethylbenzylimidazole thiolate complex) and REBTH (Ruthenocenylethene-1,2-diylbis(benzene)thiol) derivatives of Rc, which have the potential to be used as active templates for electrochemical analysis. Rc–C=C–C_6_H_4_–C_6_H_4_–SAc (REBTA) and Rc–C=C–C_6_H_4_–C_6_H_4_–SH (REBTH) are examples of Rc derivatives that were thought to be precursors for the formation of active monomolecular templates (Table 1). The aforementioned self-assembled monolayers displayed upright-oriented molecular backbones that were fastened to the substrate by atomic sulfur, revealing Rc moieties at the self-assembled monolayers-ambient interface terminal. These monomolecular templates can be of great importance for electrochemical experiments due to terminal Rc [41].

Rc undergoes electrochemically irreversible oxidation and readily forms dimers on the electrode surface. Upon oxidation to ruthenocenium, it dimerizes to bis(η^5^-cyclopentadienyl)ruthenium(II) dianion [Rc]_2_^−2^ on the electrode surface. Then, it either disproportionates to bis(η^5^-cyclopentadienyl)ruthenium(II) dication [Rc]^2+^ and decomposes to ruthenium oxide, or remains stabilized, depending on the other counter anions present in solution. Smaller anions favor dimer fragmentation, whereas larger anions stabilize the dimer and prevent its breakdown (Table 1) [27]. Rc dimers are also stabilized in the gaseous phase through noncovalent interactions. Hydrogen-bonded Rc dimers, particularly those with carboxylic acid substituents, have higher binding energies and higher dissociation barriers than amide functionalized Rc complexes. The cationic species exhibit greater stability than the corresponding anionic species. Importantly, symmetric proton transfers and energy-decomposition analyses further highlight the combined roles of electrostatic, polarization, and dispersion forces in stabilizing these assemblies [70].

**Figure 4:**
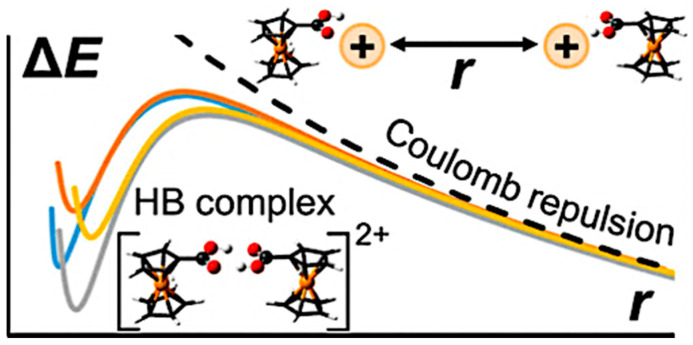
Binding energies charged hydrogen-bonded dimers of ruthenocene [70], © 2023 American Chemical Society. Adapted with permission.

In another study [35], the electrochemical oxidation of the complex Rc(η^5^-2,4-dimethyl-pentadienyl) was carried out in acetonitrile solution to produce monocationic complex [Rc(CH3CN)_3_]^+^PF_6_^−^ by cleaving the pentadienyl ligand into an organic radical that dimerized using a single electron. The stable dicationic compound, [Rc(CH_3_CN)_2(_η^3^-2,4-dimethyl-pentadienyl)]^2+^(BF_4_^−^)_2_ was created using a two-electron technique (Figure 4). The acetonitrile ligand coordinated and boosted the Ru(II) oxidation state and controlled the rate of development of the monocationic complex [71] (Figure 4). These findings demonstrate that Rc and its derivatives exhibit environmentally sensitive redox behavior, where oxidation triggers dimerization, ligand rearrangement, and solvent coordination processes that are strongly influenced by counter-anions and noncovalent interactions.

## 6. Tentative Applications of Ruthenocene in Energy Storage

**Figure 5:**
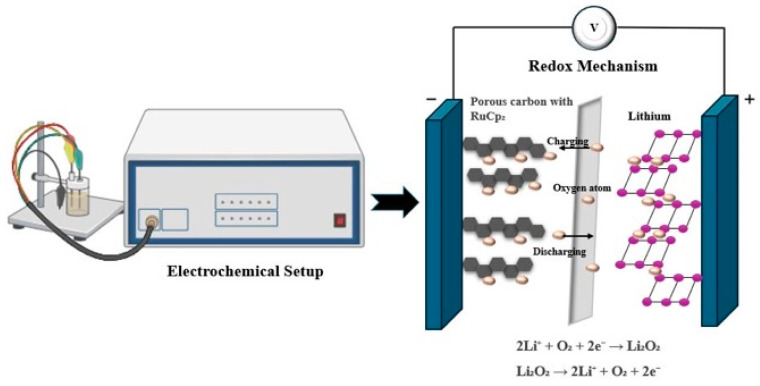
The electrochemical setup shows the redox mechanism in a lithium-oxide battery.

Current Rc-based energy storage research is largely confined to proof-of-concept studies involving Li–O_2_/Li-N_2_ batteries with activated-carbon systems, indicating that this field remains exploratory. The stability of Rc is attributed to the strong bonding between the ruthenium center and the cyclopentadienyl rings, enabling reversible electron transfer with minimal molecular degradation. Its well-defined redox cycling, reversible one-electron oxidation and reduction processes, and thermal stability make it suitable for energy-storage applications [30] (Figure 5).

The aromaticity of Rc also contributes to cyclic stability by delocalizing electrons across the ring system, making the molecule more stable overall [30,31]. Through dimerization or disproportionation events, the reactive character of the Rc^+^ cation during oxidation influences the electrochemical behavior of Rc in organic electrolytes. According to Itoi et al. [2], when Rc was hybridized through gas-phase adsorption within the micropores of microporous activated carbon with ionic liquid electrolytes, it underwent a reversible redox reaction inside the activated carbon micropores. This process increased the redox potential from 3.6 to 3.94 V (vs. Li/Li^+^) and enhanced volumetric energy density (Table 1, Figure 5) [2]. Itoi et al. [31] also highlighted that ionic liquids enhance the electrochemical oxidation of Rc. Upon oxidation in ionic liquids, the hybridized Rc molecules rearrange and contact with the carbon surface, making this system a viable option as a stable conductive interface for charge transfer processes [31] (Table 1). The presence of electron-donating boron species further enhances charge-transfer behavior and battery charging performance [62].

In Li–O_2_ batteries, Rc serves as a mobile redox mediator, lowering the charging potential to approximately 3.65 V while maintaining comparable discharge characteristics. Rc-modified Li–O_2_ batteries demonstrate improved cycling stability compared with unmodified electrodes. With a pure Ketjen black electrode, the Li–O_2_ batteries with Rc demonstrated exceptional stability and achieved 83 cycles. The charge potential plateau was significantly reduced with Rc to 3.65 V, even though the cell with Rc displayed a similar discharge voltage plateau to that without it. This suggests that Rc, as a redox mediator, can actually lower the charging potential of Li–O_2_ batteries (Table 1, Figure 5) [30].

A Ru/Mo_2_C heterostructure supported on N-doped carbon nanotubes was fabricated via atomic layer deposition, delivering high specific capacity, improved reversibility, and excellent cycling stability in Li–N_2_ batteries. The enhanced performance originates from the engineered Ru/Mo_2_C interface, which increases the density of active sites, reinforces structural stability, and optimizes electronic transfer, thereby promoting both nitrogen reduction and nitrogen evolution reactions for high-performance Li–N_2_ battery cathodes [72].

In Rc, ruthenium centers play an active role in tuning the electronic structure of electrocatalysts for biomass electrooxidation using ascorbic acid [73]. In a representative FeRu–NC system, the incorporation of trace Ru substituents results in ultralow overpotential, high current density, long-term stability, and excellent selectivity toward dehydroascorbic acid formation, thereby replacing the sluggish oxygen evolution reaction with a thermodynamically favorable two-electron oxidation process that facilitates hydrogen production [74]. These findings illustrate that Rc-based electronic modulation can be exploited to optimize structure–activity relationships in sustainable electrocatalytic systems coupled with hydrogen generation.

These studies demonstrate that Rc is a promising material for energy storage devices due to its special qualities, which include its high energy density, chemical stability and stable redox behavior. It has the potential to improve the efficiency and performance of batteries, making it a crucial area of study for next-generation energy storage research and development. Current Rc-based energy storage studies remain limited to isolated proof-of-concept systems.

## 7. Ruthenocene as Photoinitiators

Rc also behaves as a photoinitiator to catalyze polymerization reactions. When Rc interacts with an electron-accepting solvent, it forms photoactive ground-state donor–acceptor complexes. These complexes undergo charge transfer-to-solvent transitions, resulting in absorption bands in the near ultraviolet region, which, under UV radiation, undergo oxidation to form a radical cation, while the solvent is reduced to a radical anion. This photoredox process initiates anionic polymerization of the monomer (Table 1, Figure 1).

Substitution of the cyclopentadienyl rings affects the spectroscopic and photochemical behavior of Rc. The substitution of benzoyl groups on the rings shifts the absorption bands to longer wavelengths with significantly higher intensities compared to unsubstituted Rc [75]. Rc shows inert photochemical behavior in non-halogenated media; however, when combined with a suitable electron-accepting solvent, such as ethyl-2-cyanoacrylate, it forms a photoactive ground-state donor–acceptor complex. The Rc complex experiences a charge-transfer-to-solvent transition that results in a photoredox process [75]. Oviedo et al. [34] compared the two derivatives of the Rc-[60]fullerene dyad with pyrazoline and pyrrolidine groups to study charge-separated states. The Rc-pyrazolino [60]fullerene dyad experienced charge separation more efficiently than the Rc-pyrrolidino [60]fullerene dyad in benzonitrile, which may prolong the charge-separated states [34]. Cuesta et al. [40] also explored the photoinductive effect of three novel hybrid metalloporphyrin–Rc compounds. The photoinduced electron transfer occurred efficiently after photoexcitation from the Rc moiety (functioning as a donor) to the singlet excited state of the metalloporphyrins (functioning as an acceptor). The immediate binding of a pentamethylcyclopentadienyl ruthenium(II)cation [Rc]^+^ segment to several metallooctaethyl porphyrins showed optical and electrochemical characteristics that correlated with the existence of a robust electronic connection between the porphyrin core and the fused organometallic moiety. This, as a result, reverses the typical properties of metalloporphyrins in an efficient manner and offers an alternative approach for producing photovoltaic devices with many potential applications (Table 1, Figure 1) [40].

The above investigations on Rc and its derivatives sought to determine the impact of employing various methodologies. All of these investigations aimed to explore the potential of Rc-based systems to improve the stability, reactivity, and general performance of Rc derivatives in electrochemical, photoinitiator, and energy storage systems by adding various ligands and functional groups. It offers new insights into the adaptability of Rc compounds and their potential for advancement in cutting-edge technology.

## 8. Biomedical Research Based on Ruthenocene and Its Derivatives

**Figure 6:**
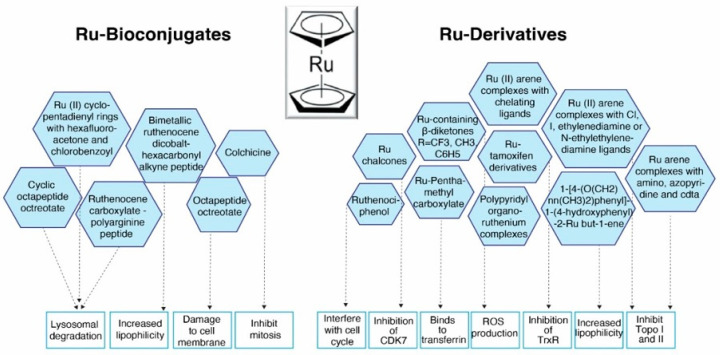
Ruthenocene and its derivatives and their anticancer mode of action.

Rc and its derivatives have received a lot of attention in biomedical research due to their unique chemical properties. Their uses include antiproliferative properties [76], photodynamic therapy (PDT) [77], photoactivated chemotherapy (PACT) [77], pharmaceutical delivery systems [78], and antibacterial [79] applications. Rc’s biocompatibility, targeting strength and overall medicinal efficacy by functionalizing it with diverse ligands and bioconjugates, has been studied by many research groups [80,81,82,83]. In particular, the anticancer properties of Rc with a special emphasis on the effects of adding different ligands to the Rc rings are presented in Table 3 and Figure 6.

### 8.1. Ruthenocene Derivatives as Anticancer Agents

**Figure 7:**
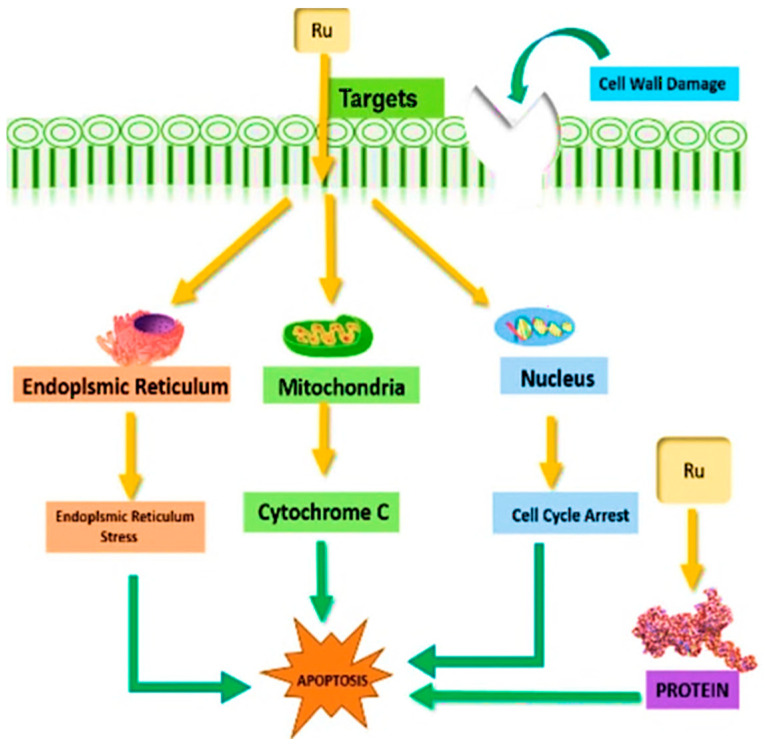
Cancer treatments based on ruthenocene complexes [97], © 2022 John Wiley & Sons. Adapted with permission.

Commonly used platinum-based chemotherapeutic agents exhibit limited specificity, often resulting in substantial toxicity to normal cells [96]. Considerable efforts have been directed toward the development of more specific anticancer agents to overcome this limitation. Many experimental studies have investigated the ability of ruthenium compounds to interact with DNA, intracellular proteins, enzymes such as carbonic anhydrase and topoisomerase I as well as key organelles such as mitochondria, the endoplasmic reticulum, and the nucleus (Figure 7). This review provides an overview of the potential use of ruthenium as chemotherapeutic agents, and highlights structure–activity relationships to support their use as anticancer ruthenium complexes in the future [97].

Rc complexes demonstrated high cytotoxicity and the effective suppression of tumor cell proliferation both in vitro and in vivo studies [76,80]. Their anticancer properties can be further enhanced through the action of chelating agents and substitutions on the Rc ring [50]. Alkyl or aryl substitution increases Rc lipophilicity, improving cellular uptake and thereby enhancing its anticancer activity [80]. Similarly, adding polar molecules to the cyclopentadienyl ring of Rc enhances its solubility and ability to bind to the cell membrane (Table 3, Figure 7) [50].

Mechanistic studies have defined the possible mode of action of the anticancer characteristics of Rc complexes and their derivatives (Figure 6). In particular, cyclometalated Ru(II) complexes exhibit higher cytotoxicity against both 2D monolayer and 3D spheroid cancer models than their polypyridyl counterparts [98]. This increased cytotoxicity may be attributed to higher hydrophobicity, improved cellular absorption, and strong DNA binding affinity [98,99,100]. The cytotoxicity of Ru(II) complexes is strongly influenced by the size and structure of polypyridyl ligands such as dipyridophenazine (dppz) and dipyridoquinazoline (dpq); larger ligands exhibit better cellular absorption and more cytotoxic effects [89]. Strong DNA associations seen in Rc complexes containing polycyclic aromatic diamines may be explained by the fact that the DNA-binding affinities of diamine ligands increase with the length of their aromatic systems. This high binding facilitates DNA thermal denaturation by increasing the generation of reactive oxygen species (ROS), as seen by the increased melting temperature and viscosity [101].

The cytotoxicity of organoruthenium complexes was positively associated with the size of the substituted ligand [78]. Polypyridyl organoruthenium complexes, in particular, have DNA binding properties that are regulated by both the size of their polypyridyl ligands and the stability of the Rc-L bond (where L = Cl, (NH_2_)_2_CS). Substitution at the chloride ligand site enhances Rc-N coordination with DNA, particularly in complexes with polypyridyl ligands of various sizes. Larger polypyridyl ligands resulted in a significant rise in DNA melting temperature and solution viscosity, indicating improved side-on intercalation and cytotoxicity [83].

Morris et al. [84] demonstrated that ligand and ring modifications significantly affect biological activity, with some Rc complexes showing potency comparable to conventional chemotherapeutics such as carboplatin. The compound 1-[4-(O(CH_2_)nN(CH_3_)_2_)phenyl] has shown efficacy against both estrogen receptor-positive (ER^+^) and estrogen receptor-negative (ER^−^) breast cancer cell lines, highlighting the importance of biomarker status in personalized therapy. In contrast, another study [86] reported that the anti-estrogenic potential of Rc derivatives in the activity of 2-ruthenocenylbut-1-ene in ER^+^ breast cancer cell lines increased with methylene chain length (n = 2–5) in ER^+^ breast cancer cell lines and surpassed that of hydroxytamoxifen; however, unlike ferrocifens, it did not inhibit ER^−^ cell proliferation. This could be due to the instability of ruthenocifen radical cations observed in electrochemical studies. These findings suggest that Rc-substituted compounds may have potential in targeted and possibly radiopharmaceutical applications for ER^+^ breast tumors [86].

The inhibitory action of Rc complexes against the human ovarian cancer cell line A2780 has been demonstrated to be regulated by substituting ligands on the arene ring such as halide, acetonitrile, isonicotinamide, ethylenediamine, or N-ethylethylenediamine [102] (Table 3, Figure 6). The most effective complexes included ethylenediamine as a chelating ligand and N-ethylethylenediamine as a monofunctional leaving group. Interestingly, inhibiting A2780 cell proliferation, the iodo analogue, and complexes containing ethylenediamine derivatives showed anticancer efficacy similar to carboplatin [84,102,103]. Mechanistically, guanine bases in DNA oligonucleotides are selectively bound by Rc complexes with reactive Rc–Cl interactions to produce monofunctional adducts [104]. It is also interesting to note that these complexes containing ethylenediamine, chlorine, N-ethylethylenediamine or iodine do not inhibit topoisomerase I or II, indicating a different mechanism of action. These results demonstrate the potential of Rc chelated arene complexes as viable options for developing metal-based anticancer agents [84,103].

The structure–activity relationships of Rc complexes bearing ligands coordinated to the ruthenium center, including amino acidates and diamines, have been extensively explored [50,88,90,92,93,94]. Complexes containing polycyclic arene and ethylenediamine ligands demonstrated superior cytotoxic efficacy against A2780 human ovarian carcinoma cells, whereas bipyridyl derivatives and complexes with polar arene substituents exhibited minimal activity [88]. The anticancer properties of β-diketonate-substituted ruthenium complexes were strongly influenced by the nature of the arene and its substituents; for example, arene and p-cymene amino acidate complexes were found to be inactive [105]. Kemp et al. [94] examined the structure–activity relationship of ruthenium complexes containing β-diketone ligands in an in vivo model and demonstrated that higher cytotoxicity was associated with decreased electron density at the ruthenium center. The pKa of the β-diketone ligands was negatively correlated with anticancer activity, whereas the oxidation potential of the ruthenium core was positively correlated [94]. In particular, substitution of the β-diketone ligand with an electron-withdrawing trifluoromethyl (CF_3_) group significantly enhanced the activity of the RcCOCH_2_COR series [106]. These findings indicate that lower ligand pKa values, higher metal oxidation potentials, and electronic modulation of the ruthenium center collectively enhance cytotoxic performance. Notably, these compounds did not display cross-resistance to cisplatin, and resistance to adriamycin was overcome by replacing ethylenediamine with 1,2-phenylenediamine [34].

Additional structure–activity studies further demonstrated efficacy against lung, pancreatic, and colon cancer cell lines (Table 3). Lee et al. [90] investigated the oxidation state of ruthenociphenol, an Rc-based organometallic analogue of ferrocifens, and showed that its cytotoxicity is closely linked to redox behavior. Upon oxidation in dichloromethane with pyridine and B(C_6_F_5_)_4_ as the supporting electrolyte, ruthenociphenol formed a (+1) cationic species capable of reversible dimerization, which subsequently generated phenoxy radicals through quinone methide intermediates; these radicals were proposed to mediate cytotoxic activity against hormone-independent breast cancer cells. In a related study, Lee et al. [92] synthesized several Rc-tamoxifen derivatives and evaluated their antiproliferative effects in breast cancer models, identifying a mono-hydroxytamoxifen-linked Rc derivative with pronounced activity (Figure 6). These findings highlight the importance of ligand design, arene substitution, and redox modulation in guiding the development of Rc-based metallodrugs.

Beyond DNA-targeting mechanisms, ruthenocenyl chalcones have also demonstrated targeted molecular inhibition. Khanapure et al. [93] evaluated the anticancer potential of ruthenocenyl chalcones and reported that these derivatives establish hydrogen bonds and aromatic interactions with cyclin-dependent kinase 7 (CDK7), indicating their potential as CDK7 inhibitors. Molecular docking studies supported this interaction, suggesting a distinct mechanism of action compared to DNA-binding Rc complexes (Figure 6).

Homo- and heterodinuclear Fc and Rc complexes incorporating 1,2,4-triazole-bis-cyclopentadienyl ligand represent another promising anticancer class of organometallic anticancer agents. The 1,2,4-triazole unit is an aromatic nitrogen-rich heterocyclic compound that functions as an efficient bridging ligand that enables efficient electronic communication between the two metal centers [107]. The Fc and Rc fragments provide high redox activity and structural robustness, whereas the bimetallic architecture often leads to higher cytotoxicity than that of mononuclear analogues due to synergistic effects involving the metal–metal interactions, improved DNA and protein interactions, and tunable redox behavior [108]. These properties make dinuclear Fc/Rc-triazole systems attractive candidates for the development of next-generation metal-based anticancer therapeutics [109]. In another study [110], ruthenocenyl analogues of etoposide, containing 1,2,3-triazolyl or aminoalkyl linkers, were produced to examine the effects of Rc substitution on anticancer activity. Replacement of the carbohydrate moiety of etoposide with a ruthenocenyl group displayed altered antiproliferative profiles, whereby the ability to interfere with important cellular targets was maintained. Biological evaluation showed that ruthenocenyl derivatives mainly affected topoisomerase II activity and the cell cycle, bringing Rc into view as a feasible organometallic scaffold for modulating the mechanism of action of known anticancer drugs [110].

The synthesis and characterization of Rc-octreotate, fluorophore-labeled derivatives, and dicobalt hexacarbonyl alkyne-functionalized octreotate were reported by Gross et al. [81]. HeLa, PT-45, and HepG2 cell lines were used to test these compounds for cytotoxicity. The usefulness of Rc as a non-toxic and adaptable label for targeted delivery in anticancer applications was confirmed by fluorescence microscopy, which showed preferential uptake of Rc–octreotate conjugates via somatostatin receptor (SSTR)-mediated pathways [81]. Similarly, the study by Wahjuni et al. [91] also showed a better cytotoxicity of pentamethylcarboxylate ruthenocene than the common chemotherapy drug cisplatin when tested for their anticancer activities against the Hela cervical carcinoma cell line.

These results highlight that ligand selection, and structural alterations are crucial for improving the anticancer activity (Figure 6). Different oxidation states and substitution patterns can have a major impact on cytotoxicity in a variety of cancer types. To maximize therapeutic potential while reducing side effects, future research should focus on optimizing these structural characteristics, especially for treating drug-resistant malignancies.

### 8.2. Ruthenocene Bioconjugates as Anticancer Agents

Bioconjugates of Rc also show great potential as anticancer drugs. By interacting with bioactive molecules like peptides, RNA, DNA, and pharmaceuticals, these substances can dramatically alter or improve the characteristics of the parent metal-based structures (Figure 6). Rc bioconjugates have been investigated as radiochemical imaging tools, medicinal agents, and heavy-atom probes in spectroscopic applications due to their versatility [111]. Although some Rc derivatives have limited stability under physiological conditions, particularly in oxidative aqueous environments, this limitation can be reduced by appropriate structural modification, which can significantly enhance their stability. However, their anticancer potential is remarkable due to their high stability in water and air as well as their strong binding affinity toward biomolecular targets.

#### 8.2.1. Ruthenocene-Nucleic Acid Bioconjugates

Rc bioconjugates incorporating peptide nucleic acid (PNA) derivatives have demonstrated broad-spectrum anticancer activity across multiple cancer cell lines. PNA is a synthetic DNA analogue in which the negatively charged sugar–phosphate backbone is replaced by a neutral N-(2-aminoethyl)glycine backbone, providing enhanced chemical stability and strong hybridization properties. In metal-based anticancer research, PNA serves as an attractive scaffold due to its analytical detectability and efficient cellular uptake [112,113]. Gross et al. [83] reported the coupling of Rc carboxylic acid peptides to the N-terminus of PNA to generate ruthenocenyl–PNA conjugates. These conjugates exhibited enhanced thermodynamic and thermal stability of PNA–DNA duplexes, suggesting that Rc labeling does not compromise DNA-binding properties but rather improves duplex stability. Such findings support the potential of Rc–PNA systems as antigene or antisense agents for anticancer applications [83].

#### 8.2.2. Ruthenocene-Peptide Targeting Conjugates

Rc has been explored in peptide-based targeting systems. Gross et al. [82] developed a heterobimetallic ruthenium–neurotensin (Rc–NT) conjugate via dicobalt hexacarbonyl alkyne functionalization followed by Rc attachment (Table 3, Figure 6). This synthetic strategy enhanced cellular uptake while overcoming solubility challenges associated with hydrophilic peptides, however, moderate cytotoxicity was observed in various cell lines such as HeLa, PT-45, and HepG2 compared to doxorubicin or cisplatin [82]. Similarly, Maschke et al. [85] introduced chlorobenzoyl and hexafluoroacetone substitutions onto the Rc cyclopentadienyl ring and subsequently synthesized bioconjugates with enkephalin, neurotensin, and fluorescein-labeled neurotensin. These derivatives demonstrated modest antiproliferative activity against the MCF-7, HT-29, and PT-45 cancer cell lines (Table 3, Figure 6). Rc conjugation has also been applied to cell-penetrating polyarginine peptides. Ruthenocenoyl–polyarginine bioconjugates exhibited enhanced binding to artificial eukaryotic membranes and increased cytotoxicity toward HeLa cells through ROS generation and apoptotic induction [95]. These findings highlight the ability of Rc to improve membrane interaction and intracellular delivery.

#### 8.2.3. Ruthenocene–Drug Hybrid System Spacing

Rc-based derivatization has also been applied to known anticancer drugs to modulate their biological activity. Ruthenocenyl–colchicine conjugates synthesized via 1,2,3-triazole linkers demonstrated 6- to 7-fold higher cytotoxicity against HepG2 liver cancer cells compared with native colchicine [80]. These derivatives induced apoptosis and exhibited broad spectrum antiproliferative effects. In vivo studies in COLO 205 colorectal cancer models confirmed submicromolar potency, indicating potential for overcoming drug resistance (Table 3). These findings suggest that Rc can modulate the mechanism of action of established chemotherapeutics without abolishing target engagement.

#### 8.2.4. Ruthenocene-Protein and Targeted Delivery Platforms

Beyond direct cytotoxicity, Rc has been explored as functional organometallic tags in medicinal chemistry. Rc-like moieties were successfully introduced into aromatic amino acid side chains of human serum transferrin without disrupting the secondary structure [114]. The modified holo-transferrin retained transferrin receptor binding capability, suggesting preserved receptor-mediated uptake This demonstrates the structural robustness and biological compatibility of Rc scaffolds for targeted delivery applications [115].

#### 8.2.5. Ruthenocene–Sulfur Amino Acid Interactions and Stability

Studies of sulfur-containing amino acid Rc amide complexes, including RM175, have shown promising antitumor activity in both in vitro and in vivo xenograft models (A2780, A2780cis, 2780AD) [77,116]. These complexes exhibit enhanced hydrogen bonding and interactions with intracellular DNA and proteins, improving stability and cytotoxicity. Rc amide complexes have also demonstrated selective interaction with sulfur-containing residues such as cysteine and methionine, potentially reducing deactivation in biological environments [87] (Figure 7).

Despite promising in vitro antiproliferative data, clinical translation of Rc-based therapeutics remains limited. Most studies are confined to cell-based assays, with insufficient investigation of pharmacokinetics, biodistribution, metabolic stability, and long-term toxicity. Additionally, Rc cytotoxicity is often multifactorial, complicating rational structure–activity optimization. While increased lipophilicity enhances cellular uptake, it may reduce selectivity and negatively affect the therapeutic index. Future development of Rc-based therapeutics will likely require the integration of targeted delivery strategies, mechanistic elucidation, and rigorous in vivo validation. Combining structural optimization with translational pharmacological studies may bridge the gap between promising preclinical findings and clinical application.

## 9. Merits, Demerits, and Their Possible Solutions

Rc complexes offer notable advantages for applications in electrochemical and biomedical fields due to their tunability, reversible redox behavior, and thermal stability. These properties support their use in energy storage, electrochemical sensing, and biological systems. Rc-controlled redox activity enables selective sensing and has been explored in targeted drug delivery, while its relatively high redox potential and favorable electron-transfer kinetics may enhance energy density and charge–discharge efficiency in electrochemical devices. However, several challenges remain. Further studies are needed to understand the long-term biological interactions, stability, and potential accumulation in tissues. Biological environments can influence Rc reactivity and stability, potentially affecting therapeutic performance. In electrochemical applications, surface by-products may lead to signal drift and reduced sensitivity, and interference from complex matrices can impact sensor selectivity. In addition, the relatively low intrinsic conductivity of Rc and material degradation under repeated redox cycling may limit performance in energy storage systems.

These limitations can be addressed through material and design strategies including the incorporation of conductive polymers or carbon-based materials, surface modification to reduce fouling, and the development of biocompatible ligands or encapsulation systems to improve stability and biological compatibility. Rc derivatives have shown promising in vitro antiproliferative activity, but clinical translation remains limited due to insufficient in vivo data and an incomplete understanding of mechanisms. Future progress will depend on integrated structure–activity studies, in vivo evaluation, scalable synthesis, and rational device and therapeutic design.

## 10. Conclusions

Ruthenocene has become a versatile organometallic scaffold with unique tunable redox properties, electronic flexibility, and a structural robustness when compared to its ferrocene analogue. This review highlights its applications in electrochemical sensing, energy storage systems, photochemical applications, and biomedical research. The redox responsive behavior of Rc is remarkably influenced by ligand environment, solvent systems, and supporting electrolytes, which allows for the formation of stable oxidation states and dimeric assemblies having functional adaptability. Rc-based systems show great potential in molecular sensing and redox labeling as well as in Li–O_2_ and Li–N_2_ battery technologies, where Rc is a highly effective redox mediator and interfacial charge transfer component. In biomedical fields, structure–activity studies of biomolecules with Rc have shown that the design of the ligand and modulation of the electronic properties determine the cytotoxicity and therapeutic selectivity of drugs and biologically active molecules. Although in vitro cell culture systems show a promising antiproliferative effect, translation to clinical development is limited due to a lack of in vivo validation and insufficient understanding of the mechanics. Future developments in Rc chemistry will require flexible ligand design, detailed study of the structure–activity relationship, material mass-production integration, and interdisciplinary concepts that can relate electrochemical studies to biological performance. Bridge filling of these gaps will be crucial to make the transition of Rc-based systems from basic research to working tutorial and clinical technology. Overall, Rc is a scientifically exciting but underdeveloped platform in terms of clinical translation, with great potential for the development of next-generation innovations stretching across the sciences of sensing, energy, and biomedical sciences.

## Figures and Tables

**Table 1:** Ruthenocene and its derivatives with various electroanalytical methods, electrode material, supporting electrolyte, and their applications.

Study	Focus	Working Electrode Material	Supporting Electrolyte (Conc.)	Electroanalytical Techniques Used
Ruthenocene				
[22]	Explored the effect of electron donation and withdrawing groups on oxidation potential of Rc	Platinum foil	Acetonitrile/0.2 M Lithium perchlorate	Chronopotentio- metric oxidation
[23]	Potentiometric titration to study effect of electron donation and withdrawing groups on the oxidation potential of Rc	Dropping Mercury/ Platinum- rotating disk	Acetonitrile/0.2 M Lithium perchlorate	Polarography/potentiometric oxidative titration
[24]	Polarographic study of Rc	Dropping mercury/ Platinum	Acetonitrile/0.1 M Tetraethylammonium tetrafluoroborate, or Tetrabutylammonium tetrafluoroborate	Polarography
[25]	Described the electrochemical behavior of Rc electrophilic specie (Rc^+^)	Platinum	Tetrabutylammonium Tetrakis(pentafluorophenyl) borate/0.1 M Dichloromethane	CV
[26]	Bis(ruthenocenium) dication undergoes a highly irreversible two-electron cathodic reaction	Glassy carbon	Dichloromethane/0.1 M Tetrakis(pentafluorophenyl) borate or Tetrakis [3,5 bis(trifluoromethyl)phenyl]borate	CV
[27]	Electrolytic effect on size of Rc anion on charge-transfer complex	Pyrolytic graphite	Sodium toluene sulfonate, 0.1 M Potassium nitrate	CV
[28]	Study the effect of change in concentration, temperature, and counter ion on Rc reversible process	Glassy carbon disk	Acetonitrile/0.1 M Tetrabutylammonium hexafluorophosphate	CV and LSV
[29]	Nucleophilic reaction with the anions of the ionic liquid media that facilitates the transfer of a second electron of electrochemically generated Rc^+^	Platinum microdisk	Acetonitrile/0.1 M Tetrabutylammonium perchlorate	CV
[30]	Improved cyclic stability of Rc for lithium oxide batteries	Ketjen black cathode	0.1 M Tetrabutylammonium perchlorate	CV and GCD
[31]	Explored the impact of the hybridization of Rc with activated carbon (AC/Rc) for energy storage devices	Carbon black	1-Ethyl-3-methylimidazolium tetrafluoroborate	CV and GCD
Ruthenocene Derivatives				
[32]	Effect of solvent and electrolyte anion on the reversible oxidation of polyRc	Glassy carbon disk	Acetonitrile/0.1 M Tetrabutylammonium perchlorate	CV
[33]	Fabrication of Rc showed irreversible redox behavior under cyclic voltammetry.	N/A	Acetonitrile/Tetrabutyl ammonium tetrafluoroborate	CV
[34]	Studied the electrochemical and fluorescence properties of Rc- based fullerene derivatives.	Glassy carbon	4:1 Dichlorobenzene/0.1 M Tetrabutylammonium perchlorate	CV
[35]	Studied effect of substitution on Rc redox potential	Glassy carbon	Acetonitrile/0.2 M Hexafluorophosphate or Lithium tetrafluoroborate	CV
[36]	Multifunctional Fc-Rc for metal– metal interactions and cation recognition properties	Platinum	1 mM Acetonitrile/Dichloromethane (3/2, *v*/*v*)/0.1 M Tetrabutylammonium hexafluorophosphate	DPV, LSV and CV
[37]	Electrochemical characterization of novel water-soluble Rc complexes	Boron doped diamond	0.1 M Potassium chloride/ Potassium hexafluorophosphate/Sodium tosylate	CV
[38]	Synthesis of multifunctional aza-substituted Rc derivatives displaying charge-transfer transitions and selective Zn(II) ions sensing properties	N/A	Dichloromethane/0.1 M Tetrabutylammonium hexafluorophosphate	CV and SWV
[39]	Electrochemical oxidation of ruthenocenyl compounds	Glassy carbon disk	Acetonitrile/0.2 M Tetrabutylammonium hexafluorophosphate	CV
[40]	Efficient photoinduced electron transfer by direct coordination of Rc metal center to metalloporphyrin produces photoexcitation. This shows the potential of metallo- porphyrin to be used in solar energy conversion	Glassy carbon	Dichloromethane/0.1 M Tetrabutylammonium hexafluorophosphate	CV
[41]	Fabrication of self-assembled mono layer templates of Rc- conjugated biphenyl ethyl thiols	Platinum	Dichloromethane/0.1 M Tetrabutylammonium hexafluorophosphate	CV
[42]	Studied the solvent and electrolyte effects in enhancing the redox activity of Rc-based complexes	Glassy carbon	Acetonitrile/0.1 M Tetrabutylammonium hexafluorophosphate	CV, LSV and SWV
[43]	Electrochemically analyzed that Rc-based chalcone with adjacent carbonyl groups are difficult to oxidize than without adjacent carbonyl group.	Glassy carbon	Acetonitrile/0.1 M Tetrabutylammonium hexafluorophosphate	CV and LSV
[44]	Electrochemically studied reduction responses of β-diketonato species in the presence of non-nucleophilic and non-coordinating supporting electrolyte.	Glassy carbon	Dichloromethane/0.1 M Tetrabutylammonium Tetrakis(pentafluorophenyl)borate	CV and LSV
[45]	Rc containing homopolymers showed improved thermal and electrochemical stability than Rc	N/A	Dichloromethane/0.1 M Tetrabutylammonium hexafluorophosphate	CV
[18]	Rc derivative acts as a redox label, studied in the presence and absence of a base	Platinum	Tetrabutylammonium/tetrafluorophenylborate	CV
[46]	Studied the catalytic activity of Rc-bound Norvaline complexes	Glassy carbon	1 mM Dimethylformamide/ 0.1 M Tetrabutylammonium hexafluorophosphate	CV
[47]	Studied the electrochemistry of Rc complexes	Glassy carbon	Dichloromethane/0.1 M Tetrabutylammonium Tetrakis(pentafluorophenyl)borate	CV
[2]	Galvanostatic properties of activated carbon/Rc hybrid electrodes in an ionic liquid electrolyte	Carbon black	1 M Sulfuric acid	CV, EIS, and GCD
[48]	Studied the change in oxidation potentials by the type and number of linker groups attached to Rc unit.	Glassy carbon	0.1 M Tetrabutylammonium hexafluorophosphate/0.1 mM Dimethylformamide carbonate	CV

Abbreviations: CV; cyclic voltammetry, LSV; linear sweep voltammetry, EIS; electrochemical impedance spectroscopy, DPV; differential pulse voltammetry, GCD; galvanostatic charge discharge, SWV; square wave voltammetry.

**Table 2:** Comparison based on the electrochemical and mechanistic characteristics of ferrocene and ruthenocene.

Parameter	Ferrocene (Fc)	Ruthenocene (Rc)	Relevance to Electrochemical Sensing
Redox couple	Fe(II)/Fe(III)	Ru(II)/Ru(III)	Rc oxidizes at higher potential
Typical oxidation potential	~0.3–0.5 V vs. Ag/AgCl	~0.6–0.8 V vs. Ag/AgCl	Rc is less susceptible to low- potential interferences
Peak separation (ΔEp)	0.06–0.10 V	0.08–0.12 V	Both quasi-reversible; Rc has slightly slower kinetics
Heterogeneous electron transfer rate constant (k^0^)	10^−3^–10^−1^ cm s^−1^	10^−4^–10^−2^ cm s^−1^ (system dependent)	Fc slightly faster ET; Rc offers better stability
Stability of oxidized form	Ferrocenium oxidizes in chloride reactions	Ruthenocenium more resistant to oxidation in chloride reactions	Rc preferred in biological fluids
Behavior in chloride media	EC-type irreversibility	Maintains reversibility	Rc superior for serum-based sensing
Tunability via ligand substitution	Moderate	High	Rc allows better redox modulation
Optical/ECL compatibility	Limited	Strong (Ru-based photo- physics)	Rc suitable for dual-mode sensing

**Table 3:** The ruthenocene derivatives and bioconjugates with different cell lines, cell viability assays, and their mode of action.

Author	Complex	Cell lines	Cell Viability Assays	Mode of Action
Ruthenocene Derivatives				
[84]	Rc complexes with chlorine, iodine, ethylene- diamine or N-ethyl ethylene-diamine ligands	A2780	Topoisomerase inhibition	Inhibition of the catalytic activity of human DNA Topo I and II
[85]	Rc with hexafluoroacetone and chlorobenzoyl	MCF-7, HT-29, PT45	Crystal violet	Endosomal entrapment, in line with the uptake mechanism of NTR entrapment in endosomes and subsequent degradation in the lysosomes
[86]	Ruthenocenylbutene complexes	MCF-7, MDA-MB-231	Methylene blue	Increased lipophilicity which increases the unspecific cell uptake by endocytosis
[87]	Rc complexes with amino, azopyridine and 1,2-cyclo hexanediaminotetraacetate	A2780, A2780AD	ATPase	Inhibition of the catalytic activity of human DNA Topo II
[88]	Rc with chelating ligands	A2780, A2780AD, HT-29, PANC-1, NX002	In vitro growth inhibition	Cross-resistance to adriamycin
[89]	Polypyridyl organo-Rc complexes	MCF-7, HT-29	Crystal violet	Increased ligand size enhances cellular uptake and lipophilicity
[90]	Ruthenociphenol	MDA-MB-231	MTS (3 h)	Instability of the quinone methide causes interference with cell cycle regulation, and increased reactivity leading to cellular damage and apoptosis
[91]	Penthamethyl carboxylate Rc	HeLa	MTS (60 h)	Transferrin-mediated Rc uptake in cells resulting in apoptosis
[92]	Rc-tamoxifen derivatives	MDA-MB-231	Methylene blue	Inhibition of thioredoxin reductase
[93]	Ruthenocenyl chalcones	MDA- MB-4355 and NCI	MTT (48 h)	Inhibit CDK7
[94]	Rc-containing β-diketones R=CF_3_, CH_3_, C_6_H_5_	HeLa, CORL2, Colo320DM, CORL23/CPR	MTT (168 hours)	ROS generation causes oxidative stress, damaging cellular components
Bioconjugates of Ruthenocene				
[82]	Bimetallic Rc dicobalt-hexacarbonyl alkyne peptide	HeLa, PT45, HepG2	Resazurin and crystal violet	Increased lipophilicity which increases the unspecific cell uptake by endocytosis
[83]	Rc bioconjugates of octapeptide octreotate	HeLa, HepG2, PT45	Resazurin and crystal violet	Damage to cell membrane, due to apoptotic or necrotic effects
[81]	Rc bioconjugates of cyclic octapeptide octreotate	HeLa, HepG2, PT45, SSTR-positive tumors	Resazurin and crystal violet	Specific uptake mechanism (SSTR receptor), entrapment inside an endosome and subsequent lysosomal degradation
[95]	Rc carboxylate— polyarginine peptide	HeLa	LMP quantification	Lysosomal degradation
[80]	Colchicine	HepG2, HCT116	Calcein accumulation	Inhibit mitosis and induction of apoptosis

Abbreviations: A2780, human ovarian cancer cell line; A2780AD, adriamycin-resistant human ovarian cells; Colo 320DM, human colorectal; COR L23, human large cell lung carcinoma; COR L2, small cell lung carcinoma; HCT116, human colon cancer cell line; Hela, human cervix epithelioid; hepG2, liver cancer cell line; HT29, human colon cancer cell line; LMP, lysosomal membrane permeabilization; MCF-7, ER-alpha-positive breast cancer cell line; MDA-MB-4355, lung cancer cell line; MDA-MB-231, ER-negative breast cancer cell line; NTRs, neurotensin receptors; NCI, lung cancer cell lines; NX002, human lung cancer cells; PANC-1, human pancreatic; PT45, pancreatic; Rc, ruthenocene; ROS, reactive oxygen species; SSTR, somatostatin receptors; Topo, topoisomerase; trxrs, thioredoxin reductases.

## Data Availability

This review article does not report any new data. All data used in this review are sourced from previously published studies and are cited accordingly.
